# A Novel Power Efficient Location-Based Cooperative Routing with Transmission Power-Upper-Limit for Wireless Sensor Networks

**DOI:** 10.3390/s130506448

**Published:** 2013-05-15

**Authors:** Juanfei Shi, Anna Calveras, Ye Cheng, Kai Liu

**Affiliations:** 1 School of Electronics and Information Engineering, Beihang University, Beijing 100191, China; E-Mails: shijuanfeifei@163.com (J.S.); liuk@buaa.edu.cn (K.L.); 2 Wireless Network Group (WNG), Universitat Politècnica de Catalunya, c./JordiGirona 1-3 Mòdul C3, Barcelona 08034, Spain; 3 i2CAT Foundation, Gran Capità 2-4 (Nexus Building), Barcelona 08034, Spain; 4 Research Center for Eco-environment Sciences, Department of Atmospheric Chemistry, Chinese Academy of Sciences, Beijing 100085, China; E-Mail: chengye0124@gmail.com

**Keywords:** wireless sensor networks, power conservation, cooperative communication, bad nodes avoidance, power efficient

## Abstract

The extensive usage of wireless sensor networks (WSNs) has led to the development of many power- and energy-efficient routing protocols. Cooperative routing in WSNs can improve performance in these types of networks. In this paper we discuss the existing proposals and we propose a routing algorithm for wireless sensor networks called Power Efficient Location-based Cooperative Routing with Transmission Power-upper-limit (PELCR-TP). The algorithm is based on the principle of minimum link power and aims to take advantage of nodes cooperation to make the link work well in WSNs with a low transmission power. In the proposed scheme, with a determined transmission power upper limit, nodes find the most appropriate next nodes and single-relay nodes with the proposed algorithm. Moreover, this proposal subtly avoids non-working nodes, because we add a Bad nodes Avoidance Strategy (BAS). Simulation results show that the proposed algorithm with BAS can significantly improve the performance in reducing the overall link power, enhancing the transmission success rate and decreasing the retransmission rate.

## 1. Introduction

Cooperative routing has been identified as an effective and useful method of reducing the negative effects of fading in Wireless Sensor Networks (WSNs). WSNs have numerous potential applications, e.g., environmental monitoring, mineral survey, traffic control and disaster response. In practical applications, a set of QoS requirements (e.g., bandwidth, transmission delay and packet loss rate) on network performance must be satisfied. However, due to the dynamic topology, time-varying wireless channel, and severe constraints on power supply, quality of service (QoS) provisioning is challenging in WSNs [1,2].

The power awareness issue is the primary concern within the domain of WSNs. As most power dissipation occurs during communication [3], routing is an important part in improving WSNs' QoS. In the same hardware conditions, a reasonable routing protocol can not only improve the quality of data transmission, but also save power and energy consumption so as to extend sensors' life-time.

For TCP/IP protocol suite in IP based architectures routing is a relevant part. Therefore, the quality of its process will affect the efficiency of the entire Internet network. Classical routing protocols in WSNs have been widely developed during these last years. In Reference [4], its authors provide an exhaustive survey on energy-efficient routing protocols for WSN as well as their classification. In Reference [5], authors present a specific classification of existent Location-based Protocols, which is the focus of this paper. In Reference [6] a survey on clustering routing protocols in WSNs is presented concluding a comparison of the existent ones. In Reference [7] they present a classification depending on network structure presenting a comparison of features and goals of data routing approaches.

The development trend of routing protocols in WSNs is that the routing protocol should save energy and power as much as possible [8,9]. What is more, it is expected to balance the amount of information transmitted by a node and at the same time to avoid reducing of the QoS. Another important aspect is that routing protocols must have security implemented, but this is out-of the scope of this work.

There still exist challenges for developing routing protocols in WSNs due to the three following reasons:

- *Smaller coverage*: mainly in short-distance communication, the general communication range is a few meters to tens of meters, so the need of transmission power is low. Because the sending power, which is the largest part of the entire transmission power consumption in wireless nodes, is growing exponentially with increasing distance, IEEE 802.15.4 protocol is fundamentally determined as a low-power agreement. The sending power in IEEE 802.15.4 is generally recommended between −3 dbm–10 dbm. With low power transmission it is difficult to ensure the quality of the transmission in a complex network environment. However, the research and development of high-power devices suitable for WSNs still takes longer.
- *Large number of sensor nodes*: it is difficult to build a global addressing scheme for large number of sensors without the high overhead maintenance. Thus traditional IP-based protocols may not be applied in WSNs. In WSNs getting data is more important than knowing the identifiers for what every node in the networks must be self-organized as the ad hoc deployment [10]. The keynote of a routing in WSNs is to establish an automatic communication mechanism for each node instead of a central deployment.
- *Topology changes*: they are a practical problem that occurs when nodes artificially or naturally fail or move. In case of topology changes, usually the new topology will not be timely informed to each node in the networks. This is seriously harmful to address -memory mode-based networks. This encounters nodes to have autonomous adaptability to failures and changes in the phenomena without any external intervention [11].

New ideas on routing in WSNs, such as cooperative routing algorithms can be used to solve the problems above to some extent. It is for that reason that they draw more and more attention in WSN research.

In WSNs, multipath fading is a great challenge [12,13]. Because of serious fading, destination nodes cannot judge the signal sent by source nodes in fading channels. In this case, in order to ensure the success of the transmission, the transmission power must be increased, which is difficult in WSNs due to the fact most nodes are battery powered and one of the main design challenges in wireless sensor networks (WSNs) is coping with resource constraints placed on individual sensor devices [14]. However, cooperation diversity is one of the ways to address decline in a favorable channel [15,16]. In recent years, more and more people began to pay attention to and research cooperative routing algorithms in WSNs. Because cooperative links can mitigate fading, achieve high spectral efficiency and improve transmission capacity for wireless networks by means of spatial diversity, and their realization is easier than that of the multiple-input multiple-output (MIMO) technique for small mobile terminals, it is theoretically possible to better adapt to the common WSNs where the node power is relatively low.

Transmit or receive diversity in cooperative routing is realized through the virtual multi-antenna array which is formed by several or all single-antennas in a network sharing each other's signal, so the basic idea of cooperation routing is that every node will have one or more cooperative relay nodes helping communication together when it is to send data to another. Each node will not only exploit its own spatial channel but also cooperative relay node's and as a result gain an additional certain spatial diversity. This inherent spatial diversity enables nodes to cooperate their communication for successful delivery to a destination [17]. The basic procedure of cooperation routing is that the source node sends a data to every node in its communication area at one time taking advantage of the broadcast nature of the wireless channel. Then, same of the receiving nodes working as the relay nodes will send the signal which has been processed to the destination node [18]. Finally, the destination node incorporates the signals sent by the source node and relay nodes according to certain rules.

At present, most cooperative routing are based on the purpose of improving the system performance on the transmission quality and efficiency. For cooperative routing research, relay node selection problem is the most important issue [19]. Currently, according to the purposes and the methods of selecting the relay node, the typical cooperative routing protocols in wireless networks can be divided into: cooperative routing protocol based on the channel quality, energy-based cooperative routing protocol, the opportunity cooperative routing protocols, distributed cooperative routing protocol, location based cooperative routing protocols, and leapfrogging strategy [20,21]. Table 1 presents the classification and comparison of cooperative routing protocols taking into account the most relevant advantages and disadvantages of each type.

*Cooperative routing protocols* based on the channel quality give full consideration to the wireless network's multi-channel characteristics. By taking into account the channel and routing selections, they make the relatively idle channel to take on more tasks during data transfer and thus reduce the transmission delay; by reducing the co-channel interference they will increase the network throughput [22]. The channel quality is indicated by the Channel Quality Indicator (CQI) which is specifically defined according to the actual situation, for example, signal-to-noise ratio (SNR), signal to interference plus noise ratio (SINR), signal and noise distortion ratio (SNDR), *etc*.

*Energy-based cooperative routing protocols* are designed to complete the data transmission with minimum energy consumption. In the MAC layer, the protocols avoid conflicts and duplication of data transmission by controlling the opening and hibernation states of the sending node and cooperative nodes thereby reducing energy consumption; in the network layer, the protocols through the optimal choice of the next hop node and cooperative nodes achieve the whole link energy minimization [23–26].

*Opportunity cooperative routing protocols* are used in the networks which do not need whole paths between source node and destination node. They used the encounter opportunities caused by the nodes movement to achieve the data transmission. They are self-organizing networks with delay and split tolerance. The opportunity network, whose nodes are not unified deployed, is different from the traditional multi-hop wireless network. Network size and nodes' initial positions have not been pre-set and the path between the source node and the destination node cannot be determined in advance.

*Distributed cooperative routing protocols* are different from the centralized routing protocol ones according to the ways of calculating and expressing control information. In selection of distributed routing nodes, the control information exchange between the nodes and the calculation of the path from the source node to the destination node is completed by each node independently, rather than by a central node.

*Location based cooperative routing protocols* assume that nodes know their location information as well as that of the surrounding nodes. Sending nodes use this location information as the basis of the selection of next nodes and relay nodes and for forwarding the data to the target area in accordance with a certain strategy, depending on the network structure [27,28].

*Leapfrogging strategy* is an improvement based on the traditional cooperative mechanism. It works in the case where the decoding of the data packet is unsuccessful at the next-hop node even after repeated retransmissions. To ensure the successful transmission of data packets, cooperative node which successfully received and decoded data packets will be as sending node to leapfrog the next-hop node which does not work well.

The remainder of the paper is organized as follows: Section 2 explains the basics of location-based cooperative routing. Then, Section 3 describes the related work. Moreover, Section 4 defines the network model of the proposed algorithm. We explain our scheme, Power-efficiency Location-based Cooperative Routing with Sensor Power-upper-limit, in detailed in Section 5. In Section 6, we list the calculation method of the simulation parameters, whereas in Section 7 we present our simulation results. Finally, Section 8 summarizes our conclusions.

## 2. Location-Based Cooperative Routing

Location-based routing has been widely hailed as the most promising approach to generally scalable wireless routing. It can enable data-directed transmission without establishing a global link state-based routing table which may cause data flooding in the entire network. It can save energy and reduce the nodes' memory demand by only storing the neighbor state information, which has good network scalability and robustness [27–29]. In our study, we fully combined the advantages of cooperative- and location-based routing protocols.

**Figure 1.**
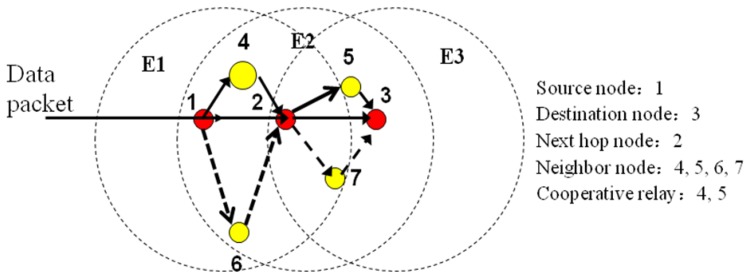
Location-based cooperative routing.

Figure 1 shows how the location-based cooperative routing works by means of an example. In this case source node 1 just needs to know the location of destination node 3 and the nodes 2, 4 and 6 in its transmission range. Firstly, node 1 will determine node 2 as the next hop node and secondly node 1 needs to choose whether node 4 or node 6 are the relay node. By a given mechanism of competition, node 1, for example, chooses node 4 as the relay node and then sends the data packet to node 2 which replaces node 1 as the source node and will do the same procedures as node 1 to send the data packet to node 3. A Request to Send/Clear to Send (RTS/CTS) handshake mechanism is used to cope with the “Hidden Stations” problem provided by IEEE 802.15.4. The “Hidden Stations” is a situation where station A and C send data to station B at the same time as A and C are not aware of each other's behavior and this causes a data conflict. Firstly, A sends RTS to B to inform B the transmission. When B receives the RTS from A, it will send CTS to all the stations in its transmission range to state that all the stations sending data to B should pause, except A. The data transmission will not begin until the handshake consummation to avoid conflicts when multiple non-visible transmission stations send a signal to the same receiving station. A retransmission request (RREQ) is sent by the data-receiving station to the data-sending station to require a retransmission when the data-receiving station cannot receive or decode the data packet in order to recover error messages in one transmission. In a message exchange network, a message is divided into several data blocks which are called data packets. A data packet will also contain the address information of the sender and receiver. These packets are then transmitted in one or more network along different paths, and reassembled at the destination. The broadcast nature of this networks means the signals sent by the transmitter can be received by all receivers that located within transmission ranges. That is the key on these types of algorithms.

## 3. Related Work

Related studies about cooperative routing in WSNs. have seen some progress during recent years. A Multi-agent Reinforcement Learning-based multi-hop mesh Cooperative Communication mechanism for wireless sensor networks (MRL-CC) [1], is a protocol with a multi-hop mesh cooperative structure. In this structure each node is implemented with coding and transmission schemes and the cooperative mechanism using a multi-agent reinforcement learning algorithm which defines the cooperative partner assignments. In the network, by considering the interactions among each other, cooperative nodes serve as multiple agents that can learn the optimal policy cooperatively by using locally observed network information and limited information exchange for reliable data disseminations. Table 2 summarizes main features of some relevant cooperative routing protocols found in the literature, and highlights the advantages and disadvantages of them.

A QoS support adaptive relay selection scheme for cooperative communications (QoS-RSCC) investigates cooperative communications for QoS provisioning in wireless sensor networks (WSNs) [2]. In QoS-RSCC, based on a multi-agent reinforcement learning algorithm, each node has an optimal relay selected from multiple relaying candidates according to packet outage probability and channel efficiency, which are part of the QoS requirements.

A novel geographic routing protocol (LCRP) that incorporates cooperative relaying and leapfrogging is proposed in Reference [29]. The concept of leapfrogging circumvention is proposed for poor radio channel conditions and aims to significantly reduce the number of retransmissions. This protocol scheme does not insist on successfully decoding a data packet at the next hop node with a sufficient number of retransmissions. Instead, it considers that there may be nodes that are further advanced towards the destination node than the next-hop node among the relay nodes which have successfully decoded the data packet in response to a RREQ from the next-hop node after the initial retransmission. In the condition of energy-constrained WSNs such an approach can potentially increase the network lifetime, yet the selections of next hop nodes and leapfrogging nodes have not been proposed in detail.

Robust Cooperative Routing Protocol (RRP) [30] is a cross-layer robust routing protocol based on node cooperation among nearby nodes for unreliable mobile WSNs. Different from the traditional routing protocols, in RRP there are several robust paths expanded from an intended path. Inside these robust paths, a reliable path is selected for packet delivery. For each packet, the robust routing protocol is capable of selecting the best path in a wide zone. Utilizing path diversity in the robust path, the intended path can easier cope with the varying topology based on the path quality, and as a consequence, the robustness against path breakage is improved.

Cooperative-Aided Routing Protocol (CARP) [31] in mobile *ad-hoc* WSNs consists of two parts as follows. The first part is to increase the operational lifetime of network by means of the decision of routing routes based on the route stability according to the mobility of mobile nodes; and the second part is to increase packet delivery ratio with advanced Signal-to-Noise Ratio (SNR) focusing on the data forwarding via the cooperative-aided routes.

Power Control based Cooperative Opportunistic Routing Protocol (PC-CORP) [32] for WSNs can ensure better data forwarding efficiency in an energy efficient manner by providing robustness to random network connectivity variations. This protocol combines the cooperative communication, sleep discipline, rendezvous scheme, and region-based routing based on a realistic radio model to model data forwarding by cross layer design in WSNs. In addition, Additive Increase Multiplicative Decrease Power Control (AIMD-PC), a lightweight transmission power control algorithm, is introduced to improve the forwarding efficiency performance and increase the robustness of the routing protocol utilizing the relay nodes' cooperation. The performance of PC-COPR is satisfying QoS requirements of application by means of investigating simulation from the perspectives of adaptation to variations in network connectivity.

An energy efficient cooperative routing scheme with space diversity called Space-Time Block Codes (STBCs) protocol [33], based on space-time bloc codes as well as the link quality, is established to improve the throughput and enhance power efficiency. In this solution, the cooperation utilizes a multiple-relay strategy as the selected multiple nodes act as multiple transmitting and receiving antennas. Power efficiency is enhanced by utilizing the full diversity available from the orthogonal STBC which can overcome multipath fading. The protocol outperforms the other two in low SNR environments and provides higher throughput and similar delays in high SNR environments compared with the traditional single-relay strategy and the single receiving diversity routing methods.

Energy-efficient Cooperative Routing Protocol (ECRP) [34,35] is a distributive implementation of the cooperative routing protocol. In this protocol, under the assumption that nodes can know the relative location of neighboring nodes, a minimal energy multi-nodes cooperative route can be found by the cooperative transmission of neighboring nodes and comparison of total power consumption. The basic form is to implement the distributive routing scheme on cooperative clusters with RREQ packets additionally carrying route power consumption information. There is a 30%–50% energy saving compared with traditional non-cooperative routing. At the same time, by trading off a little decline in energy-efficiency when using the selection strategy of cooperative nodes, the control expense and complexity of computation can fortunately be reduced.

In Reference [36], a Distance-based Energy Aware Routing (DEAR) algorithm is proposed to ensure energy efficiency and energy balancing. This algorithm is based on the theoretical analysis of different energy and traffic models. Simulation results show that compared to other routing algorithms, the DEAR algorithm can reduce the energy consumption for all sensor nodes and balance the energy distribution so network lifetime is greatly prolonged.

Most of the works mentioned have not taken into account the nodes' upper power limit that may exist for limited energy supplies and equipment strength practical application and how the networks perform at such conditions. Moreover, they also did not give much thought to the topology mutation caused by unknown bad nodes (that stop working due to energy exhaustion or damage).

In order to take advantage of the low link power and high channel gain of the cooperative routing in WSNs so that nodes can work better under extremely low power conditions, we propose a routing algorithm called Power Efficient Location-based Cooperative Routing with Transmission Power-upper-limit (PELCR-TP). Node location information analysis and selection policy based on the RTS/CTS handshaking mechanism is the core part of the algorithm. The main idea of the algorithm is that each node uses its transmit power upper limit as its transmit power in order to ensure enough transmission distance in case of low power. In this case, the transmission distance and the outage probability will mutually influence each other, both of which can be calculated knowing the lowest link power, and then the sending node will use the calculated transmission distance as the basis for selecting the location of the next hop node. The algorithm adopts a single cooperative node strategy, and the cooperative link ensures to maintain a relatively low outage probability even in a long transmission distance scenario. In addition, the algorithm further includes a bad node avoidance strategy. The cooperative node will not drop packets until the transmission of this hop success so that it can replace the next node to continue transmission when the next hop node cannot receive or decode packets.

The crucial idea of our work is to abandon the outage probability threshold which has usually been settled in some other algorithms and make outage probability as a factor in the equations when calculation the hop nodes' locations. This change makes the nodes' selections more optimum and the application range broader.

The contribution of our work is providing an algorithm which can work stably and power-efficiently with extremely low sending power. This algorithm can make WSNs awake in bad environments, for example, eco-system detection, deep-water probe, micro-sensor in military, *etc*. We will demonstrate that the proposed protocol can work stably, is power-efficient with extremely low sending power and works in “bad” environments so that we consider node density less than 0.003/m^2^, node power upper limit less than 0.00005 W, and a pass loss index of more than 3.5.

## 4. Network Model

The cooperative model used for this study takes a single cooperative node mode which means that for each hop there are only one relay node and one next hop node to transmit to. The model includes two basic link models, direct link model and cooperative link model, with some basic assumptions as described below. The transmission will automatically choose the cooperative link model when there is an appropriate relay node meeting the requirements; otherwise, a direct link model will be used.

### 4.1. Basic Assumptions

In order to evaluate the proposed algorithm, some basic assumptions are made. We assume that there are no two nodes located at the same position. All sensor nodes are equipped with the same radio transceiver. Moreover, each node only knows its own location information, power limit and channel environment parameters. Through RTS/CTS mechanism only respective location information can be transmitted between nodes, so each node just follows its own parameters to calculate and select the next hop node and relay node. Therefore, for each sending node, it seems that each subsequent node is just like itself.

The basis of the above assumptions is that usually the quality of the channel and the merits of the environment are not prone to change. That means that the changes in the propagation environment are usually smooth. Here we have chosen the path loss exponent *k* as the indicator representing merits of the network.

We use Unit Disk Graph communication model for analysis. In this model, any two nodes *i* and *j* can reliably communicate with each other if and only if:

(1) |i j|≤R

where |*i j*| is the Euclidean distance between *i* and *j* and R is the maximum transmission range

Each node in WSNs has a unique node identification number and all the links between nodes are bidirectional, *i.e.*, if there is a communication link from node *i* to *j*, there is also one from *j* to *i*.

### 4.2. Direct Link Model

**Figure 2.**
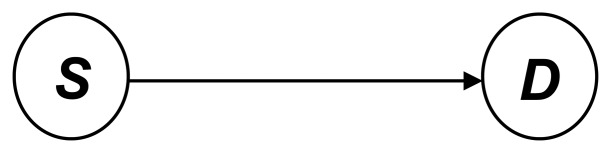
Direct link model.

The Direct link model is shown in Figure 2. The link *(S*, *D)* is composed of the sending node *S* and the receiving node *D*:

The wireless channel between a sending node *S* and receiving node *D* can be expressed by *θ* and *α*, where *θ* is the phase-shift factor, and *α* is the gain factor which equals $h_{S,D}/d_{S,D}^{k/2}$, where *k is* the path loss exponent, and *d_S,D_* is the distance between the nodes. Assume that the channel attenuation coefficient *h_S,D_* is independent and identically distributed, and subject to a Gaussian distribution with zero mean and variance equal to 1. So for the direct link, the signal received at receiver *D* is:

(2) $$\text{r}(\text{t})=\alpha_{\text{S},\text{D}}{\text{e}}^{\text{j}\theta }\text{s}(\text{t})+\text{n}(\text{t})$$

where *s*(*t*) is the transmitted signal, and *n*(*t*) is the noise signal.

### 4.3. Cooperative Link Model

**Figure 3.**
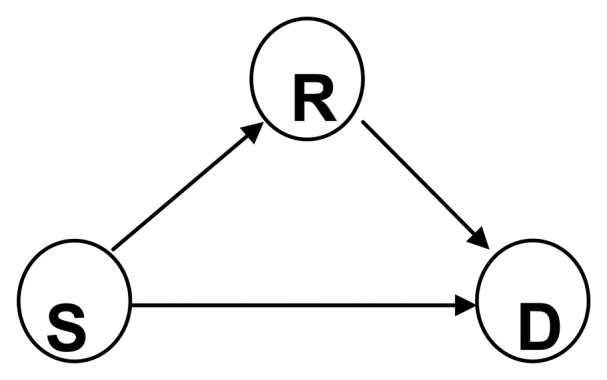
Cooperative link model

In the cooperative link model shown in Figure 3, link *S*-*D* establishes a collaborative sending mode. The collaborative link is formed by node *S* as a sender, node *R* as a cooperative node, and node *D* as a receiver. The process can be divided into two time slots. In the first time slot, the packet can be sent from source node *S* to forwarding node *D* and *R* directly. In the second time slot, the packet is sent through the relay node *R* to node *D*, and then the node *D* combines them.

Assuming that the receiver *D* both receives the data signals sent by *S* and the data signals relayed by *R* from *S*, and the transmission power *P_t_* of each node is equal for all of them, then the signal received by receiver *D* is:

(3) $$\text{r}(\text{t})=\left(\alpha_{\text{S},\text{D}}+\alpha_{\text{R},\text{D}}\right){\text{e}}^{\text{j}\theta }\text{s}\left(\text{t}\right)+\text{n}\left(\text{t}\right)$$

## 5. Pelcr-Tp Algorithm

The PELCR-TP algorithm is a location based cooperative protocol. It consists of two parts, one of which is the selection of the next hop node and the other is the bad node avoidance strategy. This algorithm is based on the principle of minimum link power. For a transmission hop, we have [37]:

(4) $$P_{S}=\frac{(2^{\mu_{0}}-1)N_{0}}{P_{D}^{\text{out}}}d^{k}$$

where *P_S_* is sending power, $P_{D}^{\text{out}}$ is outage probability and *d* is the transmission distance. In case *P_S_* is determined by the node power upper limit, *d* and $P_{D}^{\text{out}}$ have a negative correlation. Within a restricted range, we can find the optimal relation between these two parameters so that the overall link power reaches a minimum, while ensuring the success rate of the transmission. The following section describes how to select the optimal distance *d*.

### 5.1. Direct Link

For direct transmission between node *S* and *D*, the total power is [38]:

(5) $${\text{P}}_{\text{S},\text{D}}={\text{P}}_{\text{s}}+2{\text{P}}_{\text{e}}$$

where *P_e_* is the power consumed by the transmitter and 2*P_e_* counts a sending and a receiving power consumed. If the sending power has reached the maximum, the total direct power according to Equation (4) is then:

(6) $${\text{P}}_{\text{S},\text{D}}={\text{P}}_{\text{S}}^{\text{Lim}}+2P_{\text{e}}$$

where $P_{S}^{\text{Lim}}$ is the power upper limit of node *S*. The outage probability for this transmission is:

(7) $${\text{P}}_{\text{D}}^{\text{out}}=\frac{(2^{\mu_{0}}-1){\text{N}}_{0}}{{\text{P}}_{\text{S}}^{\text{Lim}}}{\text{d}}^{\text{k}}$$

As a statistical value, we can use the outage probability indirectly to indicate the expected sending times, n, in a hop as follows:

(8) $$\text{n}=\frac{1}{1-{\text{P}}_{\text{D}}^{\text{out}}}=\frac{1}{1\frac{\left(2^{\mu_{0}}-1\right){\text{N}}_{0}}{{\text{P}}_{\text{S}}^{\text{Lim}}}{\text{d}}^{\text{k}}}$$

As the node *S* just knows its own parameter information $\left(P_{S}^{\text{Lim}},k\right)$ and the location information of nodes who participate in the RTS/CTS within the transmission range, it must assume henceforth other hop conditions are equal to this hop. So in its view, the transmission distance of each hop, *d*, it is the same. *L* is the distance between *S* and *D*, so the total times, *m*, of hops is:

(9) $$\text{m}=\frac{\text{L}}{\text{d}}$$

Consequently, the total power of the link calculated by node *S* is:

(10) $${\text{P}}_{\text{total}}={\text{P}}_{\text{S},\text{D}}\times \text{m}\times \text{n}={\text{P}}_{\text{S},\text{D}}\times \frac{1}{1-\frac{\left(2^{\mu_{0}}-1\right){\text{N}}_{0}}{{\text{P}}_{\text{S}}^{\text{Lim}}}{\text{d}}^{\text{k}}}\times \frac{\text{L}}{\text{d}}$$

Let:

(11) $$\text{A}={\text{P}}_{\text{S},\text{D}}\times \text{L}$$

and:

(12) $$\text{B}=\frac{\left(2^{\mu_{0}}-1\right){\text{N}}_{0}}{{\text{P}}_{\text{S}}^{\text{Lim}}}$$

then:

(13) $${\text{P}}_{\text{total}}=\text{A}/(\text{d}-{\text{Bd}}^{\text{k}+1})$$

We take the first derivative of *P_total_*with respect to *d*, and let $\overset{\partial \text{P}}{\;}/\underset{\partial \text{d}}{\;}=0$, at which time *P_total_* reaches the only minimum value. That is:

(14) $${P^{\prime }}_{\text{Total}}=-\text{A}\left[1-\text{B}\times \left(\text{k}+1\right){\text{d}}^{\text{k}}\right]/{\left(\text{d}-{\text{Bd}}^{\text{k}+1}\right)}^{2}=0$$

And then we obtain the ideal transmission distance of this hop:

(15) $$d=k\sqrt{\frac{1}{B\times \left(k+1\right)}}=k\sqrt{\frac{1}{\frac{\left(2^{\mu_{0}}-1\right)N_{0}}{P_{S}^{\text{Lim}}}\times \left(k+1\right)}}$$

**Figure 4.**
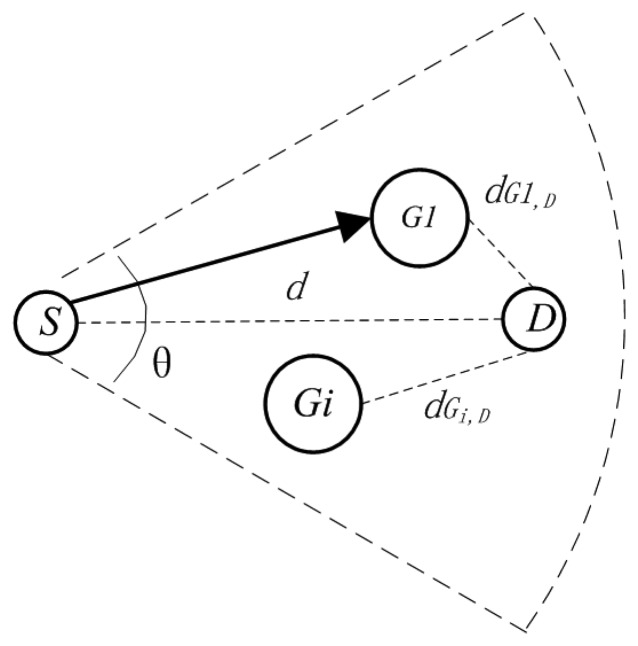
Next hop node selection.

Next-hop node's selection in direct link, shown in Figure 4, is realized by the RTS/CTS handshaking mechanism. Nodes' competition for the next hop node will use the back off time as the indicator. The back off time before the node *G_i_* replies CTS_1_ message can be formulated as:

(16) $${\text{T}}_{\text{delay}}\left(\text{i}\right)=\left[\omega {\left(\frac{{\text{d}}_{{\text{G}}_{\text{i}},{\text{D}}_{\text{i}}}}{\text{d}}\right)}^{2}+\text{R}\left(1-\omega \right){\left(\frac{1-\text{cos}\theta_{\text{i}}}{2}\right)}^{2}\right]{\text{T}}_{0}$$

where *d_Gi,Di_* is the distance between the node *G_i_* and the ideal next-hop node *D_i_*, *d* is the ideal distance, *R* (0 ≤ *R* ≤ 1) is a random number, *ω* (0 ≤ *ω* ≤ 1) is the balance factor, *θ_i_* is the angle between *G_i_* and the destination node *D* with *D_i_* as the vertex, and *T_0_* is the maximum waiting time of node *G_i_* before it forwards the message. The node whose back off time is the least will win the competition and become the next hop node. For more details readers are referred to a previous study [39].

### 5.2. Cooperative Link

#### 5.2.1. Next Hop Node Selection

Different from direct transmission, the outage probability of cooperative transmission is a comprehensive result. It is affected by the relay node *R*'s location, next hop node's location and the transmission power and so on [40]. For a single relay transmission, the determination of the location of the ideal relay node is based on the location of next hop node which, however, is unfortunately unknown, so the location of *R* must be assumed when *S* has to calculate *d*. Furthermore, it should assume *R*'s parameter information $\left(P_{S}^{\text{Lim}},k\right)$ as well.

**Figure 5.**
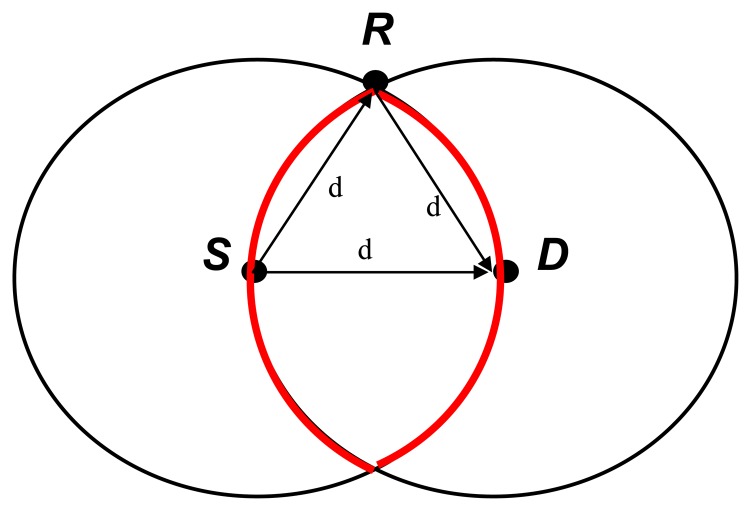
Assumption of the relay node position.

Like in direct transmission, *S* will assume that *R* has the same situation as *S* do that they have the same parameter information $\left(P_{S}^{\text{Lim}},k\right)$ and thereby *R*'s expected ideal transmission distance is also *d* [41]. In order to ensure the successful transmission, *R* must be within the sending radius of *S*, and *D* must be in the emission radius of *R*, so *R* should be in the red area shown in Figure 5. We choose the most ‘remote’ point for both *S* and *D* shown in Figure 3 as the assumed location of *R*. In this case the location of assumed *R* is the worst one in this area for relay so that hereafter wherever the final selected *R* actually is in the area it may competent to relay.

We assume that the sending and receiving processes are independent for every node. So for each group of sending and receiving processes the outage probability can be calculated according to Equation (7). For cooperative transmission, an entire hop should contain three groups of sending and receiving processes: *S* to *D*, *S* to *R* and *R* to *D*. Among these three, *S* to *R* then *R* to *D* is a continuous process, so the outage probability of an entire hop, $P_{S,R,D,}^{\text{out}}$, can be calculated as follows:

(17) $${\text{P}}_{\text{S},\text{R},\text{D}}^{\text{out}}={\text{P}}_{\text{S},\text{D}}^{\text{out}}\left[1-\left(1-{\text{P}}_{\text{S},\text{R}}^{\text{out}}\right)\left(1-{\text{P}}_{\text{R},\text{D}}^{\text{out}}\right)\right]$$

where $P_{S,R}^{\text{out}}$ and $P_{R,D}^{\text{out}}$ are the outage probabilities from *S* to *R* and *R* to *D*. As *S* assumed that ${\text{P}}_{\text{S}}={\text{P}}_{\text{R}}={\text{P}}_{\text{S}}^{\text{Lim}}$ and d_S,D_= d_S,R_ = d_R,D_ = d, according to Equation (7) we have ${\text{P}}_{\text{S},\text{R}}^{\text{out}}={\text{P}}_{\text{S},\text{R}}^{\text{out}}={\text{P}}_{\text{S},\text{R}}^{\text{out}}=\frac{(2^{\mu_{0}}-1){\text{N}}_{0}}{{\text{P}}_{\text{S}}^{\text{Lim}}}{\text{d}}^{\text{k}}$. So we can simplify the $P_{S,R,D}^{\text{out}}$ as:

(18) $$\begin{array}{c} {\text{P}}_{\text{S},\text{R},\text{D}}^{\text{out}}=\frac{\left(2^{\mu_{0}}-1\right)N_{0}}{{\text{P}}_{\text{S}}^{\text{Lim}}}{\text{d}}^{\text{k}}+2\frac{{\left(2^{\mu_{0}}-1\right)}^{2}{{\text{N}}_{0}}^{2}}{{\text{P}}_{\text{S}}^{{\text{Lim}}^{2}}}{\text{d}}^{2\text{k}}-\frac{{\left(2^{\mu_{0}}-1\right)}^{3}{{\text{N}}_{0}}^{3}}{{\text{P}}_{\text{S}}^{{\text{Lim}}^{3}}}{\text{d}}^{3\text{k}}\\ ={\text{Bd}}^{\text{k}}+2{\text{B}}^{2}{\text{d}}^{2\text{k}}-{\text{B}}^{3}{\text{d}}^{3\text{k}} \end{array}$$

where:

(19) $$\text{B}=\frac{\left(2^{\mu_{0}}-1\right){\text{N}}_{0}}{{\text{P}}_{\text{S}}^{\text{Lim}}}$$

And the expected sending times, *n*, of a hop is:

(20) $$\text{n}=\frac{1}{1-{\text{P}}_{\text{S},\text{R},\text{D}}^{\text{out}}}=\frac{1}{1-{\text{Bd}}^{\text{k}}-2{\text{B}}^{2}{\text{d}}^{2\text{k}}+{\text{B}}^{3}{\text{d}}^{3\text{k}}}$$

In a packet cooperative transmission hop, at the first time slot according to the network model described in Section 4, source node *S* broadcasts the data packet to the selected next hop forwarding node *D* and the selected relay node *R* in its communication area. Then at the second time slot, relay node *R* broadcasts the data packet received just recently to node *D* for data combination. Hence the power consumption contains two times the sending power limit, 2 sending power and 3receiving power. Consequently, the total power of an entire cooperative hop is:

(21) $${\text{P}}_{\text{S},\text{R},\text{D}}=2\times {\text{P}}_{\text{S}}^{\text{Lim}}+5{\text{P}}_{\text{e}}$$

According to Equation (10), like in direct transmission, the total power of the link calculated by node *S* is:

(22) $${\text{P}}_{\text{total}}={\text{P}}_{\text{S},\text{R},\text{D}}\times \text{m}\times \text{n}={\text{P}}_{\text{S},\text{R},\text{D}}\times \frac{1}{1-{\text{Bd}}^{\text{k}}-2{\text{B}}^{2}{\text{d}}^{2\text{k}}+{\text{B}}^{3}{\text{d}}^{3\text{k}}}\times \frac{\text{L}}{\text{d}}$$

Let:

(23) $$\text{AA}={\text{P}}_{\text{S},\text{R},\text{D}}\times \text{L}$$

then:

(24) $${\text{P}}_{\text{total}}=\text{AA}/\left(\text{d}-{\text{Bd}}^{\text{k}+1}-2{\text{B}}^{2}{\text{d}}^{2\text{k}+1}+{\text{B}}^{3}{\text{d}}^{3\text{k}+1}\right)$$

We take the first derivative of *P_total_* with respect to *d*, and let $\overset{\partial P}{\;}/\underset{d}{\;}=0$. Then:

(25) $${P^{\prime }}_{\text{total}}=-\text{AA}\left[1-\text{B}\left(\text{k}+1\right){\text{d}}^{\text{k}}-2{\text{B}}^{2}\left(2\text{k}+1\right){\text{d}}^{2\text{k}}+{\text{B}}^{3}\left(3\text{k}+1\right){\text{d}}^{3\text{k}}\right]/{\left(\text{d}-{\text{Bd}}^{\text{k}+1}-2{\text{B}}^{2}{\text{d}}^{2\text{k}+1}+{\text{B}}^{3}{\text{d}}^{3\text{k}+1}\right)}^{2}=0$$

However, Equation (25) is a transcendental equation that has no analytical solutions, so each sending node needs iterative computation. Within the values range of the actual situations, this equation must have positive roots among which the one nearest to zero is what we seek, so we use an iterative approach, whose initial value is zero, in accordance with a certain gradual step. The computation complexity of this method is linear order to d. However, empirically, subject to certain situations, the values of d are often in a small range, which makes the computation complexity nearly a constant order. Therefore we could ignore the extra power and time consumption caused by the iterative calculation [42]. More work is needed to be done to find a suitable approximate analytical solution in future research.

When determining the ideal next hop node, *S* will assume that *R* is located at the point shown in Figure 5 in order to ensure the transmission. However, the ideal relay node is not at that point. It can be derivation from Equations (7), (17) and (22) that, when the next hop node has been selected, if *R* is located in the mid-point between *S* and *D*, $P_{S,R,D}^{\text{out}}$ and *P_total_* are both the least, so we choose a mid-point between *S* and *D*as for the location of the ideal relay node and we use this ideal relay location as a reference for the selection of the actual relay node [43].

**Figure 6.**
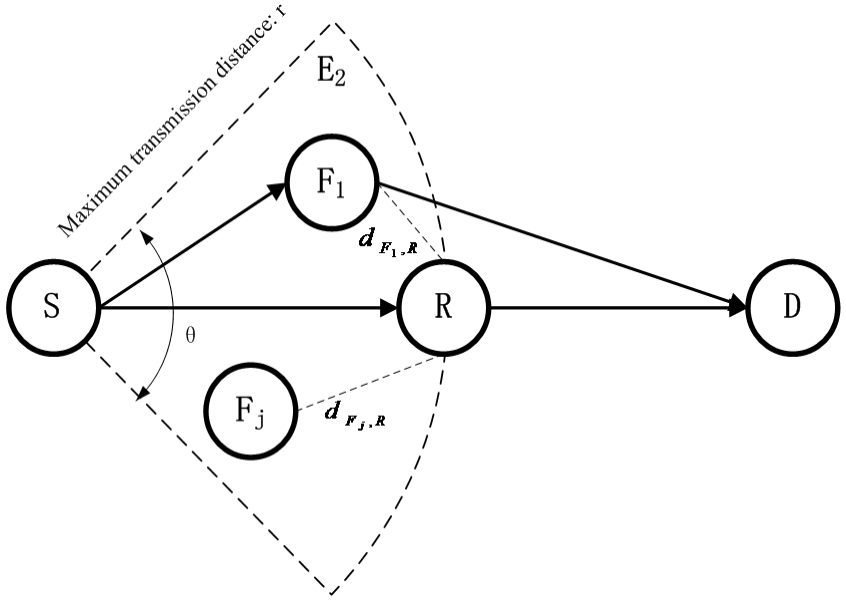
Relay node selection.

Next-hop node's and relay node's selection, shown in Figure 6, is realized by a RTS/CTS handshaking mechanism [44]. The nodes competition mechanism is the same as that mentioned in Section 5.1.

#### 5.2.2. Bad Node Avoidance Strategy (BAS)

**Figure 7.**
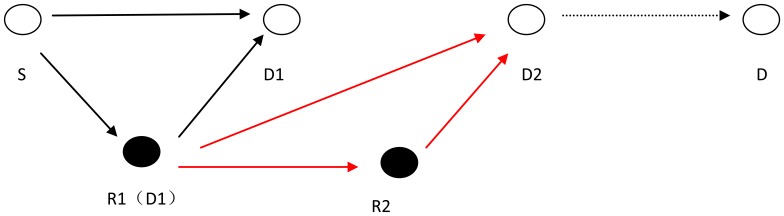
Bad node avoidance strategy.

**Figure 8.**
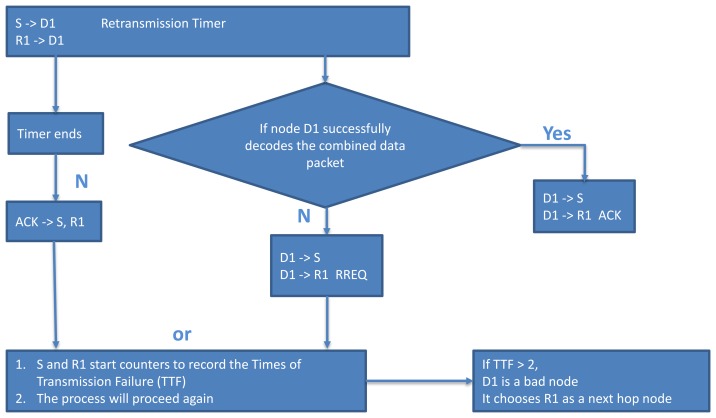
Flow chart of bad node avoidance strategy.

As shown in Figure 7, the source node *S* broadcasts the data packet to the selected next hop forwarding node *D_1_* and the selected relay node *R_1_*. In this process, when *S* or *R_1_* send the data packet, they will start retransmission timers to account for the event that the node *D_1_* cannot successfully decode the combined data packet. When the node *D_1_* could successfully decode the combined data packet from the node *S* and *R_1_*, it will send an acknowledgement packet (ACK) to *S* and *D_1_*, otherwise, it issues a RREQ to the node *S* and node *R_1_*. For *S* and *R_1_*, if the timers end without receiving the ACK or they receive the RREQ (that cancels the timer), both of them will start counters to record the Times of Transmission Failure (TTF) and the process will proceed again. However, if the times of transmission failure are more than one (TTF > 1), the forwarding node *D_1_* is believed to be a failed node. The node *S* will stop sending data packets and node *R_1_* will replace node *D_1_* as the forwarding node and continue the next hop transmission as shown in red line, so that it can reduce the times of retransmission to *D_1_*. Figure 8 shows the flow chart of bad node avoidance strategy algorithm implemented in the proposed protocol.

## 6. Performance Evaluation

We chose three parameters: total link power, transmission success rate and retransmission rate respectively; as the algorithm performance evaluation indicators [45]. Correspondingly, the average total power of an entire link is chosen to test the pros and cons of the algorithm based on the principle of minimum link power; transmission success rate is an indicator that reflects the algorithm's stability, reliability and scope of applications; and finally retransmission rate is calculated to test the algorithm's ability to respond to harsh transmission environments and bad nodes.

### Total link power

The total link power, ${\text{P}}_{\text{total}}^{\text{actual}}$, for an entire link in PELCR-TP algorithm is:

(26) $$\begin{array}{c} {\text{P}}_{\text{total}}^{\text{actual}}={\text{P}}_{\text{S},\text{D}}\left({\text{n}}_{\text{ds}}+{\text{n}}_{\text{dr}}\right)+{\text{P}}_{\text{S},\text{R},\text{D}}\left({\text{n}}_{\text{rs}}+{\text{n}}_{\text{rr}}\right)=\\ \left({\text{P}}_{\text{S}}^{\text{Lim}}+2{\text{P}}_{\text{e}}\right)\left({\text{n}}_{\text{ds}}+{\text{n}}_{\text{dr}}\right)+\left(2{\text{P}}_{\text{S}}^{\text{Lim}}+3{\text{P}}_{\text{e}}\right)\left({\text{n}}_{\text{rs}}+{\text{n}}_{\text{rr}}\right)=\\ {\text{P}}_{\text{S}}^{\text{Lim}}\left({\text{n}}_{\text{ds}}+{\text{n}}_{\text{dr}}+2{\text{n}}_{\text{rs}}+2{\text{n}}_{\text{rr}}\right)+{\text{P}}_{\text{e}}\left(2{\text{n}}_{\text{ds}}+2{\text{n}}_{\text{dr}}+3{\text{n}}_{\text{rs}}+3{\text{n}}_{\text{rr}}\right) \end{array}$$

where *n_ds_*, *n_dr_*, *n_rs_* and *n_rr_* are, respectively, the total times of first-time transmission of direct hops, retransmission of direct hops, first-time transmission of relay hops and retransmission of relay hops.

### Transmission success rate

The transmission success rate, *R_succ_*, reflecting the reliability of a link, is a kind of statistics calculated from multiple simulations. When the packet could be sent successfully from a source node to the destination, we record link success once, otherwise, link error once. The transmission success rate *R_succ_* is:

(27) $${\text{R}}_{\text{succ}}=\frac{{\text{n}}_{\text{succ}}}{{\text{n}}_{\text{succ}}+{\text{n}}_{\text{err}}}$$

where *n_succ_* is the total times of link success and *n_err_* is the total times of link error.

### Retransmission rate

Retransmission rate, *R_retrans_*, is the ratio of the times of retransmission to the times of the total transmission. This kind of statistics calculated from multiple simulations is an indicator used to test the performance of “bad node” avoidance strategy:

(28) $${\text{R}}_{\text{retrans}}=\frac{{\text{n}}_{\text{dr}}+{\text{n}}_{\text{rr}}}{{\text{n}}_{\text{ds}}+{\text{n}}_{\text{dr}}+{\text{n}}_{\text{rs}}+{\text{n}}_{\text{rr}}}$$

## 7. Simulation Results

### 7.1. Simulation Environment

In the proposed WSNs simulation environment, nodes are randomly distributed in a 500 × 500 rectangular plane area; the antenna type is omnidirectional; we use complex Gaussian white noise which variance is *N_0_* = −70 dBm; the signal bandwidth *B* = 1 MHz; balance factor *ω* = 0.78; forward angle region *θ* = 60°, the maximum waiting time *T_0_* = 200 μs in Equation (16). All the parameters above are according to the previous study [39] and the IEEE 802.15.4 protocol [46]. The source node will be located at coordinate (100, 100) and destination node at (400, 400), and then we create routes, taking the average of 1,000 different networks as the final simulation results.

**Figure 9.**
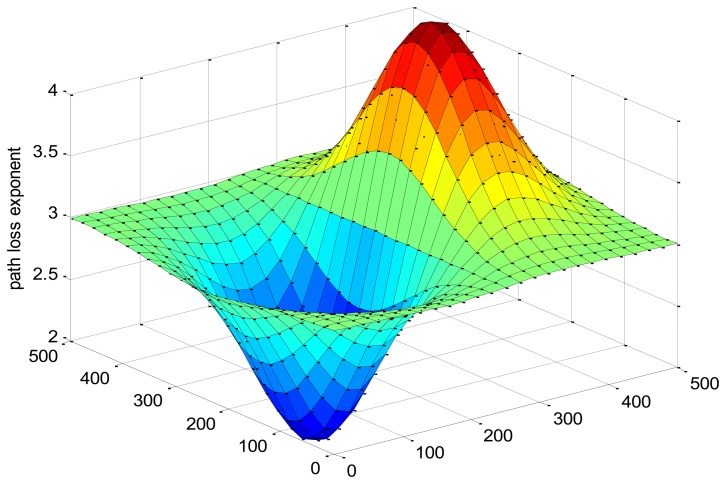
Distribution diagram of *k* in the simulation region.

When path loss *k* does not change as a control condition variable, in order to more realistically simulate the actual transmission environment, it is distributed as a fixed curved surface in the simulation area, which is shown in Figure 9 and described as:

(29) $$\text{k}=\frac{1}{2}\left(\frac{\text{x}}{125}-2\right)\times {\text{e}}^{\left(-{\left(\frac{\text{x}}{125}-2\right)}^{2}-{\left(\frac{\text{y}}{125}-2\right)}^{2}\right)}\times \text{e}+3$$

where (x, y) are the coordinate of the area. In this area, the average *k* is 3 according to the IEEE 802.15.4 protocol for low power networks. And a maxima peak exists at about (350, 250) while a minima peak at about (150, 250) in order to create a worse transmission environment area and a better one respectively to show how the algorithm works at poor and fine environments.

According to the IEEE 802.15.4 protocol, the sending power of a node is recommended from −3 dBm to 10 dBm. However, in order to test our algorithm in an extremely low power as the final aim, after multi-times simulation we get a matching sending power condition that when the sending power upper limit does not change as a control condition variable it is 0.0001 w (−10 dBm) for each node. When the bad node rate does not change as a control condition variable its value is 0.1 to all nodes. When the node density does not change as a control condition variable its value is 0.005.

### 7.2. Total Link Power

**Figure 10.**
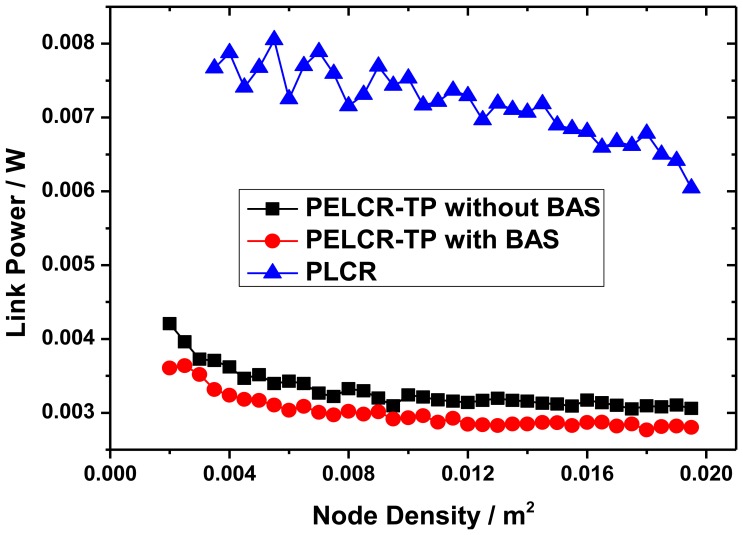
Link power *vs.* Node density.

**Figure 11.**
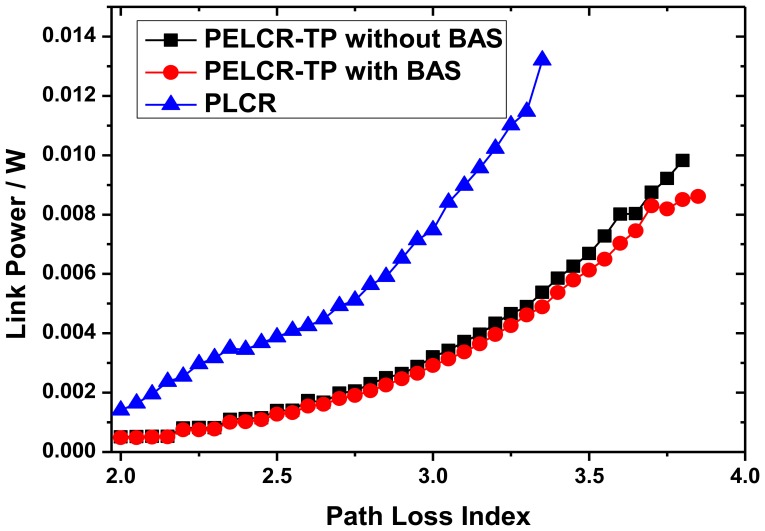
Link power *vs.* Path loss index.

**Figure 12.**
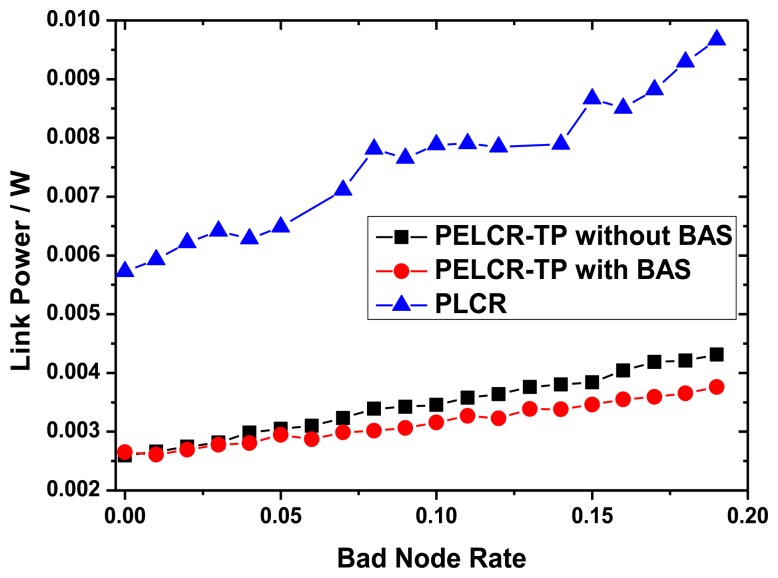
Link power *vs.* Bad node rate.

Figures 10, 11 and 12, respectively, show the comparison of the total link power of PELCR-TP algorithm with and without BAS, and PLCR algorithm at different node density, path loss index and bad node rate.

These figures show that at the same abscissa the total link power of PELCR-TP algorithm with and without BAS are both much lower than that of PLCR algorithm. That indicates in the whole variation range of node density, path loss index and bad node rate in this simulation, the PELCR-TP algorithm is much more adaptable and power-efficient. It can be seen that BAS can slightly reduce the total link power for PELCR-TP as it can overcome the “transfer resistance” and thereby reduce the total link power, which may be caused by avoiding multiple retransmissions to a bad node with poor ability to receive and decode. This part will be discussed in Section 7.4.

Figure 10 shows the impact of different node density on total link power of the three algorithms. With the increase of node density, the total link power of three kinds of routing algorithms reduce gradually, which may be caused by the fact that the next hop nodes are more and more close to the ideal next node. For PELCR-TP with and without BAS, the total link power reduces quickly in the range of node density from 0.002/m^2^ to 0.008/m^2^, while very slowly when higher than 0.008/m^2^, which indicates the PELCR-TP algorithm has the ability to determine the appropriate next node without being influenced by the node density even when the density is still low. On contrast, the total link power of PLCR is continuously reducing, whose curves's undulation is also caused by the low node density in which condition there is a significantly uncertainty of the distance between nodes.

Figure 11 shows the impact of different path loss index on total link power of the three algorithms. With the increasing of path loss index, the total link power of three kinds of routing algorithms increase exponentially as the sending power is proportional to the *d^k^*, where *d* is the transmission distance. The slope of the increasing total link power of PLCR algorithm with path loss index is much sharper than that of both PELCR-TP algorithms with and without BAS. That indicates that PELCR-TP algorithm is more suitable in poor transmission environment whose path loss index is relatively high.

Figure 12 shows the impact of different bad node rate on total link power of the three algorithms. With the increasing of bad node rate, the total link power of three kinds of routing algorithms increase gradually for the increasing of the retransmission times caused by “bad nodes”.

In summary, though the node density and power upper limit value are relatively low, as well the path loss index and the bad node rate are relatively high, which means the transmission condition is relatively poor, PELCR-TP algorithm has a more outstanding performance in the saving power consumption than PLCR algorithm. In addition, the BAS can contribute to link power saving to a certain extent.

### 7.3. Transmission Success Rate

**Figure 13.**
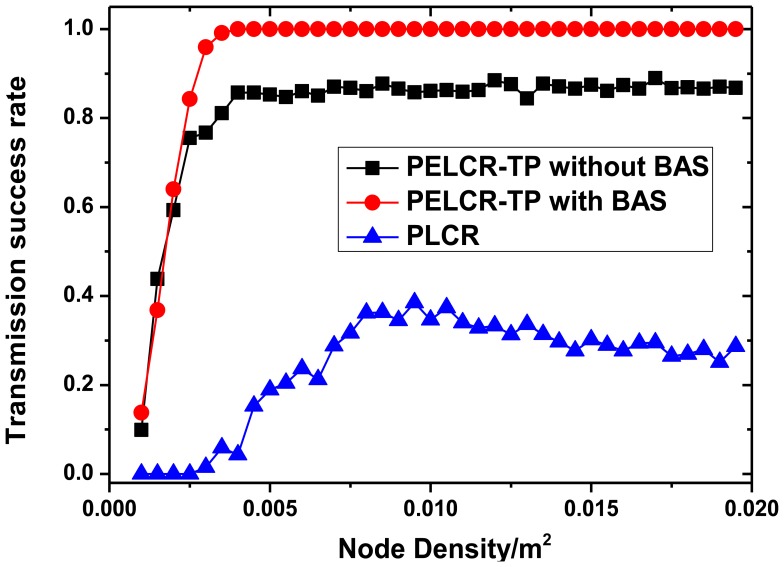
Transmission success rate *vs.* Node density.

**Figure 14.**
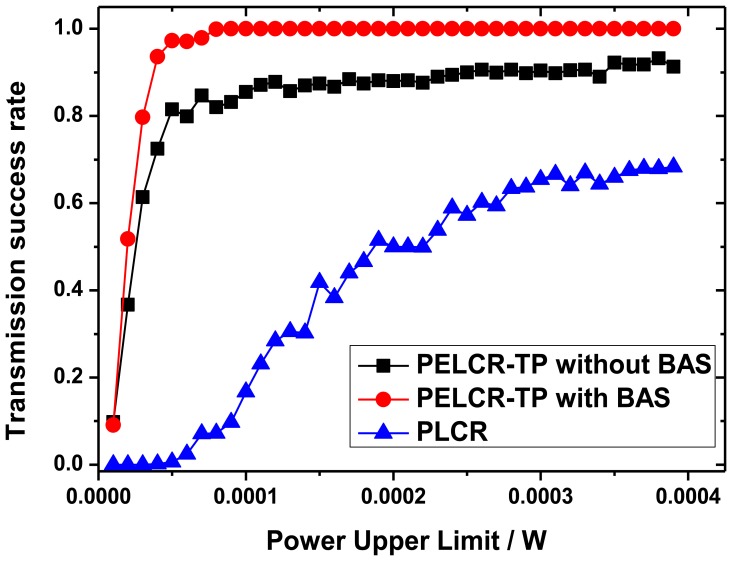
Transmission success rate *vs.* Power upper limit.

**Figure 15.**
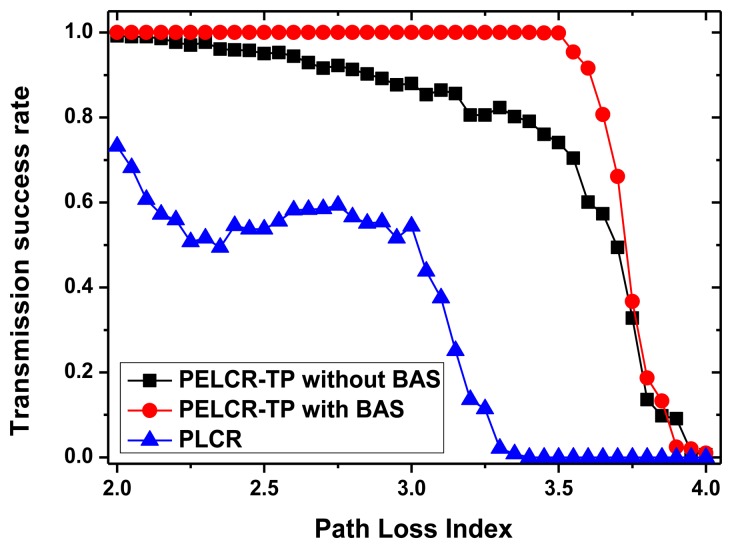
Transmission success rate *vs.* Path loss index.

**Figure 16.**
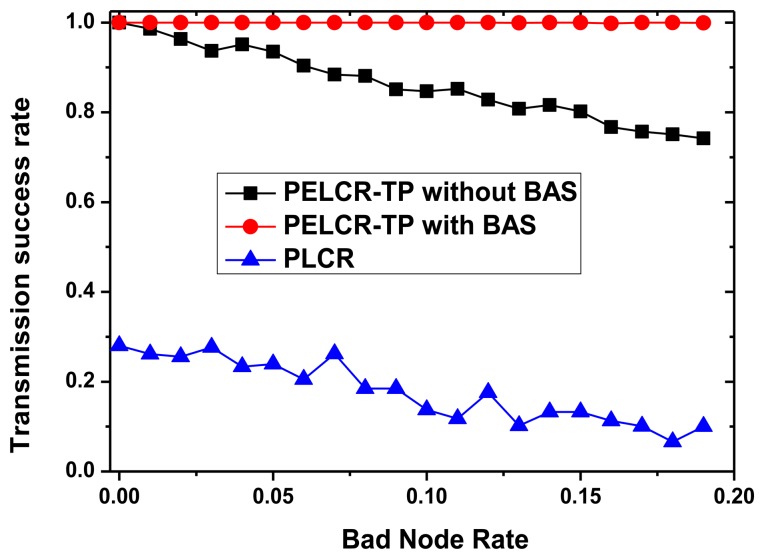
Transmission success rate *vs.* Bad node rate.

Figures 13, 14, 15 and 16, respectively, show the comparison of the transmission success rate of PELCR-TP algorithm with and without BAS, and PLCR algorithm at different node density, power upper limit, path loss index and bad node rate.

It can be seen from Figures 13, 14, 15 and 16 that in the vast majority of the range of the abscissas, the transmission success rate of PELCR-TP algorithm with and without BAS are both much higher and more stable than that of PLCR algorithm. The transmission success rate of PELCR-TP algorithm with BAS shows a very steady value approaching 1.0, a higher level than that without BAS.

Figure 13 shows the impact of different node density on transmission success rate of the three algorithms. There is an obvious inflection point both in PELCR-TP with and without BAS when the node density is around 0.003/m^2^. When the density is larger than this point, the transmission success rate of PELCR-TP algorithm shows a stable and level trend, while a sharp drop when the density is smaller than that point. In addition the inflection point of PLCR appears around 0.008/m^2^. The sharp drop of transmission success rate occurs when the average maximum transmission distance nodes can provide in this condition is shorter than the average distance between the nodes, so the value of the inflection point can indirectly reflect the ability of an algorithm that the maximum transmission distance nodes can provide, which can be calculated from Equation (7). This indicates that compared with PLCR algorithm, PELCR-TP algorithm can transmit farther under the same conditions.

The inflection points like in Figure 13 also appear in Figure 14, which is likewise caused by insufficient transmission distance according to Equation (7) when the sending power upper limit is very low. However, the difference is that for PELCR-TP algorithm without BAS there is a slope when power upper limit is larger than the inflection point. As this slope does not occur in PELCR-TP algorithm with BAS, it may be caused by the bad nodes' influence. In addition, there is no obvious inflection point for PLCR algorithm.

Figure 15 shows the impact of different path loss index on transmission success rate of the three algorithms. Only in the PELCR-TP algorithm with BAS an obvious inflection point appears where *k* is around 3.5. The reason for this point can be also attributed to the insufficient transmission distance according to Equation (7) when path loss index is high. The difference between PELCR-TP algorithm with and without BAS, that there is an obvious slope for the latter when *k* is smaller than 3.5, may indicate that when the power upper limit is low (0.0001 w) and path loss index is high, according to Equation (8), the outage probability for each hop will increase and create some “bad nodes”. Here we need to mention that when the path loss index is near 2.0, the transmission success rates of PELCR-TP algorithm with and without BAS, are almost equal and approaching 1.0. This phenomenon can be explained as follows: when the value of path loss index is near 2.0, the transmission environment is close to the ideal environment and the hop number in a whole link will be few, according to Equations (8) and (10); as the destination node cannot be set up as a bad node, it will reduce the ratio of bad nodes in the entire link. This can also be used to explain the drop of transmission success rate of PLCR algorithm where *k* goes from 2.0 to 2.3.

Figure 16 shows the impact of different bad node rates on transmission success rate of the three algorithms. Except for the PELCR-TP algorithm with BAS, the transmission success rates of both the other two algorithms have a negative linear relationship with bad node rate. In summary, when node density and sending power upper limit are relatively low, while path loss index and bad node rate are relatively high, the PELCR-TP algorithm with BAS seems more competent with a very stable and reliable performance in transmission success rate until the appearance of the inflection point.

### 7.4. Retransmission Rate

**Figure 17.**
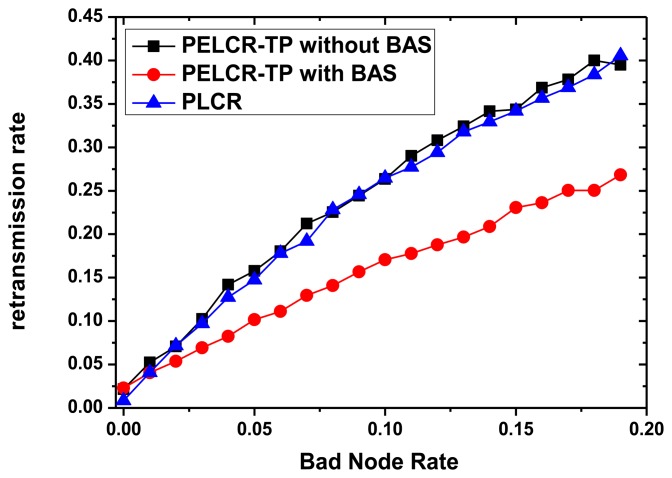
Retransmission rate *vs.* Bad node density.

Figure 17 shows the comparison of the retransmission rate of PELCR-TP algorithm with and without BAS, and PLCR algorithm at different bad node rate.

It can be seen from Figure 17 that with the increasing bad node rate the retransmission rates of the three algorithms increase. The slope of the PELCR-TP algorithm with BAS is much lower than that of both the PELCR-TP algorithm without BAS and PLCR algorithm, while the latter two have no obvious differences between each other. This indicates that in the transmission condition of this simulation, BAS effectively avoids multiple retransmissions to bad nodes and thereby reduces the retransmission rate, but the PELCR-TP algorithm itself does not affect the retransmission rate.

### 7.5. Path Nodes

**Figure 18.**
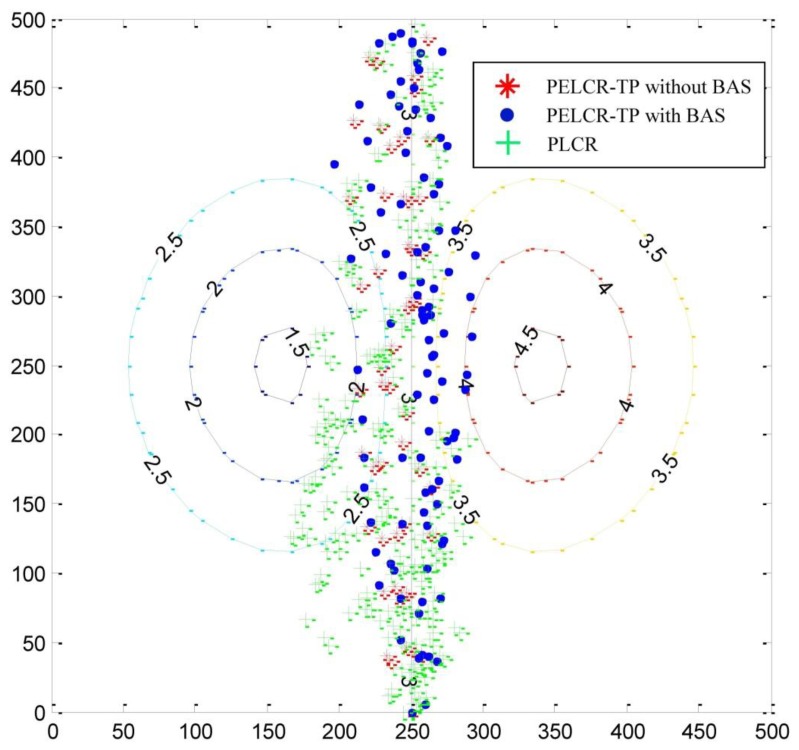
Pathways node maps.

Figure 18 randomly shows 10 path node results of the PELCR-TP algorithm with and without BAS, and the PLCR algorithm at different bad node rates from 1,000 simulations in which the power upper limit is 0.0001 w, bad note rate is 0.1 and the node density is 0.005. The source node and destination node are located at (250, 0) and (250, 500). The middle axis coincides with the line which represents *k* = 3. The area on the left of the middle axis is the region with a better transmission environment whose *k* is lower, while the other side is the region with a worse transmission environment whose *k* is higher. The path nodes of the PELCR-TP algorithm with BAS are symmetrically distributed on both sides of the middle axis. On contrast, for the other two algorithms the path nodes on the left side of the middle axis are more than on the right. This indicates that the PELCR-TP algorithm with BAS can work well wherever *k* is higher or lower with the help of BAS. The path nodes of PLCR algorithm is more concentrated near the source node than that near the destination node, while the path nodes of PELCR-TP algorithm with and without BAS both have symmetrically vertical distributions. This means the new algorithm has more reliable performance with few failures. This figure visually displays the reliable performance of the PELCR-TP algorithm and the high adaptability BAS can provide.

## 8. Conclusions and Future Work

In this paper we propose a novel location-based cooperative routing algorithm for WSNs, named PELCR-TP, that takes advantage of the low link power and high channel gain of the cooperative routing in WSNs and make it work well in the case where the transmission power is really low. Node location information analysis and selection policy based on the RTS/CTS handshaking mechanism is the core part of the algorithm. The main idea of the algorithm is that each node uses its transmit power upper limit as its transmit power in order to ensure enough transmission distance in case of low energy. In this case, the transmission distance and the outage probability, both of which can be calculated under the lowest link power, will mutually influence each other, and then the sending node will use the calculated transmission distance as the basis for selecting the location of the next hop node. The algorithm adopts a single cooperative node strategy, and the cooperative link ensures a relatively low outage probability is maintained, even for a long transmission distance scenario. In addition, the algorithm further includes a bad node avoidance strategy. The cooperative node will not drop packets until the transmission of this hop success so that it can replace the next node to continue transmission when the next hop node cannot receive or decode packets.

Simulation results show the following conclusions: firstly, when nodes' transmission power upper limit is extremely low (*i.e.*, 10^−5^ to 4 × 10^−4^) and the path loss index and bad node rate are relatively high, 2–4 and 0–0.2 respectively, PELCR-TP could significantly reduce the overall power and retransmission rate and enhance the transmission success rate, compared with PLCR, the algorithm proposed in a previous study. Secondly, the PELCR-TP algorithm shows a very stable performance over a fairly large range of conditions, as the transmission success rate is approaching 1.0 until an obvious inflection point appears. Thirdly, the retransmission rate will be lower and transmission success rate will be higher with Bad node Avoidance Strategy (BAS) than without. This shows that PELCR-TP algorithm with BAS can better adapt to the WSNs with low node density, small transmission power and bad transmission environment.

As future work we expect to find a way to make this routing algorithm not only power-efficient, but also energy-efficient, for example, by some energy awareness strategies as proposed in Reference [47]. Other performance metrics such as coverage, QoS, or security will be taken into account and optimized by the way of, for instance, the proposals presented in [48–50]. What is more, we should research the approximate calculation of the transmission distance. Last but not least, we want to study the implementation when the routing algorithm adopts the multi-relay strategy. All above are aimed to improve the performance of WSNs.

## Figures and Tables

**Table 1.** Classification and comparison of cooperative routing protocols.

**Type**	**Advantages**	**Disadvantages**
Channel quality-based	No multi-node resource allocation problems	Gain incremental decreases with the increasing of the number of relay nodes while the link cost increases
Energy-based	Simultaneously reduce power consumption and energy consumption without no loss of QoS	Little coexistence between the efficiency of the overall link power and the fairness among nodes
Opportunity	Ability to respond to random changes on network topology	Hard to ensure the selected path with feasible minimum power, energy consumption, and path length
Distributed	Suitable for Ad Hoc networks and WSNs without a central information node	Challenge in getting nodes location information
Location based	No need of the central node and a global location information table	Uneven distribution of the workload among nodes; Cannot cope with topology changes
Leapfrogging strategy	Good response to the link interruption	Not suitable for multi-cooperation networks

**Table 2.** Comparison of cooperative routing protocols.

**Routing Algorithm**	**Features**	**Advantages**	**Disadvantages**
**LCRP**	Enables cooperative relaying in an on-demand manner, and takes into account both location and channel state information for next-hop selection	↑ Reduces the number of transmissions required to reach a destination ↑ Saves energy and increase the network lifetime	↓ It does not consider the scenario that topology mutation
**RRP**	Uses the cooperative nodes within the transmission range as buffers to cope with path breakage	↑ Improves robustness while achieving considerable energy efficiency	↓ It is not good at reducing the transmission delay
**CARP**	Stable routing routes and takes advantage of cooperative-aided data transmission	↑ Increases the operational routes lifetime ↑ Increases packet delivery ratio with advanced SNR	↓ It needs more transmission power
**ECRP**	A minimum energy multi-nodes cooperative path is constructed by the cooperative transmission of neighboring nodes and comparison of total power consumption	↑ Improves energy-saving performance greatly	↓ It needs a good channel environment
**MRL-CC**	Multiple agents can cooperatively learn the optimal policy by using locally observed network information and limited information exchange	↑ The network system is more stable; ↑ The ability to reduce the delay at higher network traffic load	↓ The uniformity of the load among the nodes to be improved
**QoS-RSCC**	The optimal relay selection policy is learned collaboratively by the routers from a series of trial-and-error interactions with the dynamic network, without the needs of prior knowledge of the network model and centralized control	↑ Steady reduction in delay. ↑ Cooperative diversity gain with channel utilization efficiency	↓ It needs a good channel environment
**PC-CORP**	Combines the region-based routing, rendezvous scheme, sleep discipline and cooperative communication to model data forwarding by cross layer design in WSN	↑ Strong stability in response to topology changes	↓ Requires higher transmission power when the number of nodes is large
**STBCs**	Multi- relay strategy where the selected multiple nodes act as multiple transmitting and receiving antennas	↑ Higher throughput and similar delay in high SNR environments	↓ The total energy consumption of the system is high
**DEAR**	All the intermediate nodes will consume their energy at similar rate, which maximizes network lifetime	↑ Fairness among nodes. ↑ Lifetime is greatly prolonged	↓ Requires the precise location of the nodes which need more energy
